# Inaugural seizure in a patient submitted to electroconvulsive therapy and anti-psychotic treatment: Who’s the culprit?

**DOI:** 10.1192/j.eurpsy.2021.879

**Published:** 2021-08-13

**Authors:** I. Figueiredo, A.C. Rodrigues, I. Pereira, C. Oliveira, A. Bento

**Affiliations:** 1 Clínica 3, Centro Hospitalar Psiquiátrico de Lisboa, Lisboa, Portugal; 2 Unidade De Reabilitação, Centro Hospitalar Psiquiátrico de Lisboa, Lisboa, Portugal; 3 Clínica 4 - Unidade De Alcoologia E Novas Dependências, Centro Hospitalar Psiquiátrico de Lisboa, Lisboa, Portugal

**Keywords:** Epiletic seizure, ECT, major depressive disorder

## Abstract

**Introduction:**

Electroconvulsive Therapy (ECT) is one of the most effective treatments for Depressive Disorder. Although its safety and tolerability have been throughout the years, it still holds common mild and rarely persistent side effects.

**Objectives:**

The aim is to review some of the most recent data on the connection between inaugural seizures in psychiatric patients being submitted to ECT for treatment of Major Depressive Disorder, while also discussing the possible contribution of the concomitant use of clozapine and clomipramine.

**Methods:**

The authors present a case report of an episode of an inaugural seizure in a patient submitted to ECT, with concomitant use of clozapine and clomipramine. A search on Pubmed and Clinicalkey was performed, from which the relevant publications were selected and reviewed.

**Results:**

The authors present a 62 year old woman who developed an inaugural generalized tonic-clonic seizure after being submitted to ECT for treatment of Recurrent Major Depressive Disorder (RMDD), while also carrying out clozapine and clomipramine dosage reduction, with the purpose of discontinuation. The patient had no history of previous seizures, nor were there relevant findings in the patient’s neurological examination, blood work, brain CT or EEG.

**Conclusions:**

There is a plethora of possible factors involved in the development of an inaugural seizure. Although, the risk of spontaneous seizure during ECT is low, it may be increased by the concomitant use of drugs which can lower the seizure threshold. In most cases, when ECT was resumed after removal of such triggers, there were no further complications.

